# Association between body mass index and breast cancer intrinsic subtypes in Japanese women

**DOI:** 10.3892/etm.2012.621

**Published:** 2012-06-25

**Authors:** KOSEI KIMURA, SATORU TANAKA, MITSUHIKO IWAMOTO, HIROYA FUJIOKA, YUKO TAKAHASHI, NAYUKO SATOU, KAZUHISA UCHIYAMA

**Affiliations:** Section of Breast and Endocrine Surgery, Department of General and Gastroenterological Surgery, Osaka Medical College, Osaka 569-8686, Japan

**Keywords:** breast cancer, intrinsic subtype, body mass index, case-only analysis, Japanese population

## Abstract

The purpose of this study was to examine the association between body mass index (BMI) and breast cancer intrinsic subtypes in Japanese women. A more complete understanding of the subtypes of breast cancer may elucidate the mechanisms affecting the etiology and mortality associated with each subtype. Tumor data on 531 invasive breast cancer cases subtyped by estrogen receptor, progesterone receptor and human epidermal growth factor receptor 2 (Her2) status were obtained [luminal A, luminal B, triple-negative (TN) and Her2-type]. Demographics (age at diagnosis, menopausal status and BMI) were collected from medical records. Case-only odds ratios (ORs) and 95% confidence intervals (CIs) were estimated using logistic regression, adjusting for age at diagnosis. Of the 531 cases, 333 (62.7%) were luminal A, 85 (16.0%) were luminal B, 43 (8.1%) were Her2-type and 70 (13.2%) were TN. Compared with luminal A cases, premenopausal TN cases were more likely to be obese (OR, 4.11; 95% CI, 1.10–14.40), while postmenopausal TN cases were more likely to be underweight (OR, 3.14; 95% CI, 1.19–8.01). Premenopausal luminal B cases were likely to be underweight or obese, while luminal B and Her2-type cases were likely to be underweight. In the present study, significant heterogeneity of associations between BMI and tumor subtypes was observed. Breast cancer subtypes may have various etiologies associated with each subtype.

## Introduction

Among women in Japan, the incidence rate of breast cancer has increased sharply during the past three decades and this cancer is now the most prevalent malignancy among women (1). This increase may be accounted for by the changing prevalence of Japanese women with the established anthropometric or reproductive risk factors (2).

Breast cancer is characterized by its molecular and clinical heterogeneity. The analysis of gene expression arrays and immunohistochemical (IHC) markers has classified breast cancers into the following five intrinsic subtypes (3,4): luminal A [estrogen receptor (ER)- and/or progesterone receptor (PR)-positive, human epidermal growth factor receptor 2 (Her2)-negative and low Ki-67 labeling index (<14%), a marker of cell proliferation], luminal B (ER- and/or PR-positive, Her2-negative and high Ki-67, or ER and/or PR-positive, Her2-positive and any Ki-67), Her2-type (ER- and PR-negative, and Her2-positive), basal-like [ER- and PR-negative, Her2-negative, cytokeratin (CK) 5/6-positive and/or epidermal growth factor receptor (EGFR)-positive] and unclassified tumors. Approximately 80% of triple-negative (TN) tumors (ER- and PR-negative, and Her2-negative) express basal markers, resulting in the TN subtype usually being used as a surrogate marker for the basal-like subtype (5,6). If a reliable Ki-67 measurement is not available, an alternative assessment of tumor proliferation including histological grade (HG) may be used to distinguish subtypes (6).

Luminal tumors have been associated with the most favorable prognoses, while Her2-type and basal-like tumors, or their surrogate TN tumors, have been associated with a poor outcome (7–11). These subgroups may represent distinct etiologies of breast cancer; they certainly have important implications for therapy and effectiveness, and influence recurrence and mortality risk (12).

A number of epidemiological studies have reported that African-American ethnicity, younger age at diagnosis, higher body mass index (BMI) and higher waist-to-hip ratio (WHR) were all associated with the TN subtype versus luminal A subtype (5,12–15). An analysis of the Polish Breast Cancer Study found that early age at menarche and the highest BMI among premenopausal women were associated with basal-like disease, whereas elevated BMI decreased the risk of luminal A tumors in premenopausal women (15). By contrast, Phipps *et al* reported that an increased BMI was associated with luminal and TN tumors, but only among women not currently on hormone therapy (14).

However, it remains uncertain whether these associations also occur in Japanese populations, since only a few Japanese studies have been conducted (16,17). In addition, the prevalence of obesity, defined as a BMI of 30 kg/m^2^ or more, is less in Japan than in Western countries. Therefore, additional research is required. The purpose of the present study was to examine the associations between BMI and breast cancer subtypes separately in premenopausal and postmenopausal Japanese women. A more complete understanding of the subtypes of breast cancer, particularly the TN subtype, may elucidate mechanisms affecting etiology and mortality associated with this aggressive, difficult to treat disease.

## Materials and methods

### Study population and data collection

The present study population consisted of 632 patients diagnosed pathologically with breast cancer at Osaka Medical College (Osaka, Japan) between 2006 and 2011. Bilateral incident breast cancer (n=35), male breast cancer (n=6) and ductal or lobular carcinoma *in situ* (n=60) were excluded, resulting in 531 cases available for the present study.

Age at breast cancer diagnosis, ethnicity, menopausal status and self-reported height and weight around the time of diagnosis were obtained for all cases from the medical records. BMI was calculated as the weight in kilograms divided by the square of patient height in meters (kg/m^2^). Written informed consent was obtained from all patients and this study was approved by our Institutional Review Board (Osaka Medical College, Osaka, Japan).

### Classification of tumor subtypes

For all breast surgical or core needle biopsy specimens, the ER and PR status and Her2 expression were determined by IHC (ER, SP1, PR, 1E2, Her2/neu, 4B5; Ventana, Denver, CO, USA). The cut-off point for positivity for ER and PR in breast tumors was 10%. Her2 status was assessed by Dako HercepTest (Dako, Carpinteria, CA, USA). Tumors were considered Her2-positive in cases of 3+ staining intensity. All tumors with 2+ staining were analyzed by fluorescence *in situ* hybridization (FISH). If the FISH score (Her2:17 cen) was greater than 2.0, the tumor was considered to be Her2-positive. These IHC and FISH assays were performed at the Pathology & Cytology Laboratories (PCL Japan, Saitama, Japan).

Since the Ki-67 labeling index was measured only for certain cases, tumor proliferation was assessed using HG (6). Therefore, tumor subtype was classified into the following four types using ER, PR, Her2 status and HG. Cases that were ER- and/or PR-positive, Her2-negative and HG 1 or 2 were classified as luminal A cancers; cases that were either i) ER- and/or PR-positive, and Her2-positive or ii) ER- and/or PR-positive, Her2-negative, and HG 3 were classified as luminal B; cases that were ER- and PR-negative and Her2-positive were classified as Her2-type; and cases that were ER- and PR-negative and Her2-negative were classified as TN cancers.

### Covariate classification

The demographic and anthropometric covariates of interest were classified as follows; age at diagnosis (<50, 50–64, ≥65 years), menopausal status (pre, post) and BMI (<18.5, 18.5–24.9, ≥25 kg/m^2^) based on the WHO classification (18).

### Statistical analysis

The comparisons of patient demographics with breast cancer subtypes were conducted using Pearson Chi-square tests. Case-only analyses using disease subtypes are a useful exploratory tool to uncover etiological heterogeneity (19). This may ultimately lead to an improved understanding of why certain breast cancer patients present with a more aggressive disease, and therefore, are more likely to succumb. The case-only odds ratios (ORs) and 95% confidence intervals (CIs) were estimated using logistic regression as a measure of association, as implemented in JMP version 9 (SAS Institute, Cary, NC, USA). The ORs were calculated among cases only using luminal A, the most common subtype, as the comparison group. All models were adjusted for age at diagnosis except when this covariate was the predictor of interest. CIs not overlapping with 1.00 or P<0.05 were considered to indicate a statistically significant result.

## Results

### Distribution of breast cancer subtypes

In all cases, the patients were Japanese. The distribution of breast cancer tumor subtypes in this study is presented in Table I. Of the 531 cases with IHC marker data, 333 (62.7%) were classified as luminal A, 85 (16.0%) were luminal B, 43 (8.1%) were Her2-type and the remaining 70 cases (13.2%) were TN. The distribution of patient demographics (age at diagnosis, menopausal status and BMI) did not differ significantly with breast cancer tumor subtype.

### Case-only odds ratios

Case-only ORs comparing each subtype to luminal A are presented in Table II and are adjusted for age at diagnosis. Of the TN cases, postmenopausal TN cases were more likely to be underweight (OR, 3.14; 95% CI, 1.19–8.01). Although certain epidemiological studies have reported that a higher BMI was associated with premenopausal TN cases compared with luminal A cases (5,13), this association was not found among premenopausal TN cases analyzed using BMI 18.5–24.9 kg/m^2^ as the reference in this analysis. However, there were no underweight cases (BMI 18.5 kg/m^2^) among the premenopausal TN cases in the present study. Therefore, the association between BMI and the TN subtype was also analyzed using BMI <25 kg/m^2^ as the reference (Table III). Compared with luminal A cases, premenopausal TN cases were more likely to be obese (OR, 4.11; 95% CI, 1.10–14.40), similar to studies from Western countries (5,12,13).

Compared with luminal A cases, premenopausal luminal B cases were likely to be underweight (OR, 3.27; 95% CI, 0.88–11.39) or obese (≥25 kg/m^2^; OR, 3.32; 95% CI, 0.98–10.81), yet this association was of borderline significance.

Compared with luminal A cases, luminal B and Her2-type cases were likely to be underweight (BMI <18.5 kg/m^2^), yet this association was of borderline significance (luminal B: OR, 2.12; 95% CI, 0.97–4.46; Her2-type: OR, 2.53; 95% CI, 0.92–6.36).

## Discussion

In the present analysis of 531 breast cancer cases in Japan, associations between breast cancer subtypes and BMI were examined. Compared with luminal A cases, premenopausal TN cases were significantly more likely to be obese, while postmenopausal TN cases were significantly more likely to be underweight. Premenopausal and postmenopausal luminal B and Her2-type cases tended to be underweight compared with luminal A cases, while premenopausal luminal B cases tended to be underweight or obese.

The present results among premenopausal TN cases are consistent with other studies (5,13). Kwan *et al* reported that premenopausal TN cases were more likely to be overweight and/or obese at diagnosis compared with luminal A (5). Although we were unable to further classify TN cases into basal-like and unclassified, similar to the results of the Carolina Breast Cancer Study (CBCS; case-case analysis) (13), the present premenopausal TN cases tended to have higher BMIs. The CBCS reported that an elevated WHR was associated with a strong increase in the risk of basal-like breast cancer among pre- and postmenopausal women, and an inverse association between elevated BMI and luminal A breast cancer was observed among premenopausal women, but no association was demonstrated for basal-like breast cancer in a case-control study (13). Other case-control studies also reported that obesity was associated with the risk of premenopausal TN breast cancer (14,15).

The conflicting results for elevated BMI between premenopausal TN and luminal A tumors are consistent with hormonally associated factors that affect luminal tumors more strongly. Obesity among premenopausal women has been associated with lower estrogen levels, possibly due to irregular menstruation and anovulation (20), which may explain why elevated BMI increased the risk of TN tumors. Obesity is also correlated with hyperinsulinemia and insulin resistance. Insulin resistance has been hypothesized to increase breast cancer risk in premenopausal women via increased mitotic activity and enhanced cell proliferation in breast epithelial tissue (21). Furthermore, overexpression of the leptin receptor is found in breast tumors with a high grade, a feature associated with TN breast cancer (22). A higher BMI in patients with premenopausal TN breast cancer may be associated with these factors.

Previous epidemiological studies have shown a consistent association between elevated central adiposity and increased breast cancer risk in postmenopausal women (23), and the latest large-scale systematic review and meta-analysis of prospective observational studies has reported a positive association between obesity and postmenopausal breast cancer risk (relative risk, 1.12; 95% CI, 1.08–1.16) (24). Certain Japanese epidemiological studies have reported that this association may be limited to ER-positive breast cancer in Japanese populations (16,17). In the present study, postmenopausal TN cases were significantly more likely to be underweight compared with luminal A cases, which is in agreement with these associations in Japanese populations. The mechanism behind this increase in postmenopausal luminal breast cancer risk may reflect an increase in the concentration of bioavailable estradiol, which results in turn from an increase in estrogen production by aromatase in adipose tissue (25,26).

By contrast, the results of certain Western studies are different from the results of Japanese studies. In the CBCS, WHR, which is an index of the relative accumulation of abdominal versus gluteal fat, was used as a measure of abdominal adiposity, and a positive association between elevated WHR and breast cancer risk was observed among both postmenopausal luminal A and basal-like breast cancer (luminal A: OR, 1.8; 95% CI, 1.3–2.6; basal-like: OR, 2.7, 95% CI, 1.3–5.4) (13). The Washington State study (case-control study) reported that, among women not currently on menopausal hormone therapy, BMI was associated with a risk of luminal tumors (OR, 1.7; 95% CI, 1.2–2.4) and tended to be associated with a risk of TN tumors (OR, 2.7; 95% CI, 1.0–7.5) (14). Trivers *et al* reported that an inverse association with BMI was observed among luminal tumors, but no effect of BMI was observed among ER- and PR-negative tumors in the case-control study (12).

Discrepant results among studies may have arisen due to differences in sample sizes, study populations, laboratory methodologies and different classifications for breast cancer subtypes. In particular, Japanese populations are extremely different from Western populations. Although the prevalence of BMI over 30 kg/m^2^ in Western studies is approximately 30% (12–14), that of BMI over 30 kg/m^2^ in the present study was only 6%, similar to another Japanese study (3.2%) (16). We suggest that this difference in study populations may be responsible for the discrepant results.

In the present study, premenopausal luminal B cases tended to be not only underweight but also obese compared with luminal A cases. These associations suggest obesity-related and nonobesity-related mechanisms. Suzuki *et al* have proposed two-dimensional mechanisms, namely obesity-related and lean-related biological mechanisms (17). The obesity-related mechanisms include irregular menstruation and anovulation due to premenopausal obesity (17). These conditions may decrease exposure to ovarian hormones, which may decrease the risk of luminal breast cancer. Furthermore, overexpression of the leptin receptor, which is related to obesity, is found in breast tumors with a high grade (22). These mechanisms may cause the association between luminal B cases and obesity. By contrast, lean-related mechanisms include various vital roles of the mammary fat pad in normal mammary gland morphogenesis (27,28), possibly in close conjunction with other hormones, including estrogens and progesterone (29). Since progesterone may stimulate body fat deposition (30), a low BMI in early adulthood may indicate an insufficient mammary fat pad or progesterone deficiency, predisposing to breast cancer in later life (31). The progression of mammary epithelial cells from undifferentiated ER-negative mammary cells to differentiated cells may be linked to tumor subtypes (32). These mechanisms may result in the association between luminal B cases and being underweight. Among luminal cases, keeping an optimum weight in early adulthood may be important to prevent luminal B breast cancer, which has a poorer prognosis than luminal A cancer.

Although the present results tend to be in agreement with those of other studies, the limitations of this study should be discussed. The first is that the study population of 531 women diagnosed with invasive breast cancer was smaller than that of other epidemiological studies. However, the majority of Western studies did not effectively investigate Asian populations. Only BMI, age at diagnosis and menopausal status were analyzed. Further analysis of other factors (family history of breast cancer, reproductive history, breastfeeding, smoking, alcohol use or hormone use) should be conducted. In the present study, only case-only comparisons were conducted, and it must be emphasized that the associations reported here are all in reference to the risk of having a luminal A tumor and should not be extended to the risk of having invasive breast cancer. However, case-only analysis among tumor subtypes is a useful exploratory tool to examine etiological heterogeneity among the subtypes (19). Finally, there were no data on CK5/6 and EGFR tumor markers to further classify TN cases into basal-like and unclassified.

In summary, using a case-only analysis to assess the associations between BMI and breast cancer subtypes (luminal A, luminal B, TN and Her2-type), significant heterogeneity of associations by tumor subtype was observed. Compared with luminal A cases, premenopausal TN cases are more likely to be obese, while postmenopausal TN cases are more likely to be underweight. Maintaining the optimal weight may be useful to consider in order to prevent TN breast cancer. In addition, maintaining the optimal weight in early adulthood may be important to prevent luminal B breast cancer. In conclusion, breast cancer intrinsic subtypes may have different etiologies associated with each subtype.

## Figures and Tables

**Table I:** Distribution of breast cancer tumor subtypes and patient demographics (n=531).

Patient demographics	Luminal A n=333 (62.7%) n^a^ (%)^b^	Luminal B n=85 (16.0%) n^a^ (%)^b^	TN n=70 (13.2%) n^a^ (%)^b^	Her2-type n=43 (8.1%) n^a^ (%)^b^	P-value^c^
Ethnicity					
Asian	333 (100.0)	85 (100.0)	70 (100.0)	43 (100.0)	
Other	0 (0.0)	0 (0.0)	0 (0.0)	0 (0.0)	
Age at diagnosis, years					0.549
<50	87 (26.1)	21 (24.7)	15 (21.4)	6 (14.0)	
50–64	126 (37.8)	32 (37.6)	26 (37.1)	22 (51.2)	
≥65	120 (36.0)	32 (37.6)	29 (41.4)	15 (34.9)	
Mean (± standard deviation)	59.3 (12.7)	58.7 (12.2)	60.6 (13.9)	61.4 (8.6)	
Menopausal status					0.321
Premenopausal	95 (28.5)	23 (27.1)	16 (22.9)	7 (16.3)	
Postmenopausal	238 (71.5)	62 (72.9)	54 (77.1)	36 (83.7)	
BMI (kg/m^2^)					0.289
<18.5	24 (7.2)	12 (14.1)	9 (12.9)	7 (16.3)	
18.5–24.9	220 (66.1)	53 (62.4)	42 (60.0)	26 (60.5)	
≥25	89 (26.7)	20 (23.5)	19 (27.1)	10 (23.3)	

a Numbers are observed numbers (not adjusted for sampling probabilities).

b Percentages are adjusted for sampling probabilities.

c From Pearson Chi-square test across cancer subtypes. TN, triple negative; BMI, body mass index.

**Table II:** Case-only odds ratios (ORs) and 95% confidence intervals (CIs) from logistic regression models of associations between breast cancer tumor subtypes and demographics.

	Luminal A (comparison)	Luminal B			TN			Her2-type		
Patient demographics	n	n	OR	95% CI	n	OR	95% CI	n	OR	95% CI
Age at diagnosis, years										
<50	87	21	0.91	0.48–1.67	15	0.71	0.35–1.39	6	0.55	0.19–1.42
50–64	126	32	0.95	0.55–1.65	26	0.85	0.47–1.53	22	1.40	0.70–2.87
≥65	120	32	Ref		29	Ref		15	Ref	
BMI (kg/m^2^)										
<18.5	24	12	2.12	0.97–4.46	9	1.99	0.82–4.47	7	2.53	0.92–6.36
18.5–24.9	220	53	Ref		42	Ref		26	Ref	
≥25	89	20	0.91	0.50–1.60	19	1.07	0.58–1.94	10	0.84	0.37–1.79
BMI (kg/m^2^) premenopausal										
<18.5	10	5	3.27	0.88–11.39	0			2	2.96	0.35–18.25
18.5–24.9	72	12	Ref		11	Ref		4	Ref	
≥25	13	6	3.32	0.98–10.81	5	2.94	0.80–10.04	1	1.23	0.06–9.76
BMI (kg/m^2^) postmenopausal										
<18.5	14	7	2.10	0.74–5.60	9	3.14	1.19–8.01	5	2.22	0.64–6.70
18.5–24.9	148	41	Ref		31	Ref		22	Ref	
≥25	76	14	0.66	0.33–1.26	14	0.88	0.43–1.73	9	0.81	0.34–1.80

TN, triple negative; BMI, body mass index; Ref, reference group.

**Table III:** Case-only odds ratios (ORs) and 95% confidence intervals (CIs) from logistic regression models of associations between TN subtype and BMI.

	Luminal A (comparison)	TN		
Patient demographics	n	n	OR	95% CI
BMI (kg/m^2^)				
<25	24	51	Ref	
25–29.9	220	16	1.05	0.55–1.94
≥30	89	3	0.72	0.16–2.22
BMI (kg/m^2^) premenopausal				
<25	82	11	Ref	
25–29.9	10	5	4.11	1.10–14.40
≥30	3	0	-	-
BMI (kg/m^2^) postmenopausal				
<25	162	40	Ref	
25–29.9	60	11	0.74	0.34–1.50
≥30	16	3	0.76	0.17–2.43

TN, triple negative; BMI, body mass index; Ref, reference group.
